# Prevalence of High-Risk Pregnancies in an Urban Field Practice Area of Mumbai, India: A Community-Based Analytical Cross-Sectional Study

**DOI:** 10.7759/cureus.108249

**Published:** 2026-05-04

**Authors:** Shwetangi R Shinde, Devashri Ganorkar, Nidhi P Sastry

**Affiliations:** 1 Community Medicine, Lokmanya Tilak Municipal Medical College and General Hospital, Mumbai, IND

**Keywords:** anaemia, antenatal care, caesarean section, high-risk pregnancy, maternal health, urban slum area

## Abstract

Introduction

A high-risk pregnancy (HRP) is defined as a condition in which the mother, foetus, or both are at increased risk of adverse outcomes compared to a normal pregnancy or the general population. However, there is limited data available from urban slum settings for effective health planning and resource allocation for the management of HRPs. The current study aims to estimate the prevalence of HRPs in an urban slum of Mumbai and to identify the sociodemographic and obstetric factors and delivery characteristics associated with HRPs.

Methodology

An analytical cross-sectional study was conducted from January to December 2023 in an urban slum field practice area of a tertiary care institute in Mumbai. A total of 292 recently delivered women (within two months of delivery) were selected using systematic sampling. Data were collected through a pre-validated case record form and verified using medical records. HRPs were identified using the Pradhan Mantri Surakshit Matritva Abhiyan (PMSMA) criteria. Sociodemographic, obstetric, and delivery-related variables were analysed. Associations were assessed using Chi-square/Fisher’s exact test and multivariable logistic regression.

Results

The prevalence of HRP was 56.5% (n = 165), with 39.5% (n = 115) having a single risk factor and 17.1% (n = 50) having multiple risk factors. Severe anaemia and previous caesarean section were the most common risk factors (n = 40, 24.2% each). On bivariate analysis, increasing maternal age (p < 0.001), no schooling (p = 0.03), nuclear family type (p = 0.01), multiparity (p < 0.001), and private sector registration (p < 0.001) were significantly associated with HRP. On multivariable analysis, multiparity was independently associated with HRP (AOR: 1.85; 95% CI: 1.07-3.20, p = 0.03). HRPs were significantly associated with higher preterm delivery (n = 20, 12.1% vs. n = 6, 4.7%; p = 0.04), caesarean section (n = 92, 55.8% vs. n = 30, 23.6%; p < 0.001), neonatal morbidity (n = 64, 38.8% vs. n = 27, 21.3%; p = 0.002), higher out-of-pocket expenditure (OOPE) (median INR 6000 vs. 4000; p = 0.009), and longer hospital stay (median 4 vs. 3 days; p < 0.001). Early initiation of breastfeeding was significantly lower among HRPs (n = 81, 49.1% vs. n = 102, 80.3%; p < 0.001).

Conclusion

HRPs are highly prevalent in urban slum settings and are associated with adverse maternal and neonatal outcomes, as well as an increased economic burden. Strengthening early identification, addressing modifiable risk factors such as anaemia, evaluating rising caesarean section rates, and improving access to affordable maternal healthcare are essential to reduce the burden of HRPs.

## Introduction

A high-risk pregnancy (HRP) is defined as a condition in which the mother, foetus, or both are at increased risk of adverse outcomes compared to a normal pregnancy or the general population [1]. Identifying such pregnancies is an important part of maternal health programs, particularly in low-resource settings [2].

Globally, the burden of HRPs varies widely, ranging from 19% to over 75%, depending on the setting and criteria used [3-6]. Studies from Mexico (19.1%), Ethiopia (26.4%), the Democratic Republic of Congo (33.1%), and Brazil have highlighted anaemia, hypertension, previous caesarean section (CS), and extremes of maternal age as key contributors [4,6-8]. Similar patterns are observed in Nepal, with prevalence ranging from 14% to 33%, where maternal age, parity, and socio-economic factors play significant roles [5,9,10]. In India, national-level estimates from the National Family Health Survey (NFHS-5) indicate that nearly 49.4% of pregnancies are high-risk, with short birth spacing, previous adverse birth outcomes, and previous CS being key risk factors [11]. Maharashtra itself reports a high burden (47.7%), with higher prevalence among socioeconomically disadvantaged groups [11].

The epidemiological profile of HRPs is evolving due to emerging risk factors. Delayed childbearing has increased the risks of chromosomal abnormalities, hypertensive disorders, preeclampsia, gestational diabetes mellitus, preterm deliveries, and CS rates [12]. With the broader rise in non-communicable diseases (NCDs), hypertensive disorders of pregnancy continue to be a major contributor to maternal mortality and morbidity [13]. Simultaneously, CS rates have been increasing globally, often disproportionately among HRPs [11,14]. In India, the rates have tripled from NFHS-2 (7.1%) to NFHS-5 (21.5%), with a sharp increase between NFHS-3 and NFHS-4 [15]. These increasing rates have led to a larger pool of women with previous CS deliveries, predisposing them to repeat interventions [16]. While CS is lifesaving when medically indicated, unnecessary procedures contribute to increased healthcare costs and morbidity [17].

Given the severe, life-threatening maternal and foetal complications, as well as the out-of-pocket expenditure (OOPE) associated with HRPs, their identification is not just a clinical tool but also an important managerial asset for resource allocation, staff training, patient education, quality improvement, and policy development aimed at improving pregnancy outcomes. Most of the existing literature referenced above (nationally and internationally) focuses on rural areas. Health information systems like the Health Management Information System (HMIS) and Reproductive and Child Health (RCH) portals collect important information [18,19]. However, HMIS data are facility-based and do not provide community-level insights, while the RCH portal collects data on only two HRP conditions - severe anaemia and pregnancy-induced hypertension. Hence, there is limited information for effective health planning and resource allocation for the management of HRPs in urban slums. Therefore, the current study aims to estimate the prevalence of HRPs in an urban field practice area of a tertiary care institute in Mumbai and to identify the sociodemographic and obstetric factors, as well as delivery characteristics, associated with HRPs.

## Materials and methods

Study setting

The study was conducted in an urban slum of Mumbai, which also serves as the field practice area of a tertiary care institute. This area is home to approximately 127,000 residents, predominantly from lower- and middle-socioeconomic groups.

Timeline and study design

The study was an analytical cross-sectional study conducted over a one-year period, from January 1, 2023, to December 31, 2023.

Study population

Recently delivered women are systematically enlisted in the Postnatal Care (PNC) and Immunization registers at the local Health and Wellness Centre (HWC), in addition to their registration with Accredited Social Health Activists (ASHA) and Community Health Volunteers (CHV). Women who experienced a live birth in the preceding two months were included to minimise recall bias and to ensure that they are likely to have all pertinent documents related to their pregnancy.

Sampling

The sample size was calculated using the formula for a cross-sectional study with finite population correction $n = DEFF * \frac{[N z^{2}pq]}{[d^{2}(N-1)+z^{2}pq]}$. The population of the study setting was 126,973. Based on the crude birth rate for the district (9.33 per 1,000 population in 2022), obtained from the local HWC, the expected number of pregnancies was estimated as ((9.33/1000) × 1,26,973), with an additional 10% adjustment for pregnancy wastage, yielding approximately 1,303 pregnancies per year, which constituted the finite population. The prevalence of HRP in India is reported to range from 20% to 30% in population-level studies [2]. Assuming an average prevalence of 25%, with a 95% confidence interval, 5% absolute allowable error, and a design effect of 1.2 based on NFHS-5 [15], the minimum required sample size was 292.

The sampling technique employed was systematic sampling: sampling interval $k = \frac{N}{n}$, where, N = 1303, n = 292. Hence, k = 4.46 ~ 4. From the list of eligible women, every fourth woman was selected. If the selected woman did not consent to participate in the study, the next woman from the list was approached until the sample size was attained.

Variables and measurement

The study included socio-demographic, obstetric, and clinical variables. Socio-demographic variables comprised age groups, religion, years of schooling, socioeconomic status, and type of family. Obstetric variables included parity, number of living children, inter-pregnancy interval, pregnancy registration status, and, among registered pregnancies, the place and trimester of registration. HRPs were classified using the Pradhan Mantri Surakshit Matritva Abhiyan (PMSMA) criteria [2]. Delivery-related variables included time, mode, and place of delivery, birth weight of the newborn, early initiation of breastfeeding, maternal and neonatal complications, length of hospital stay, and OOPE.

Operational definitions

Years of schooling were categorised as per NFHS-5 [15], and socioeconomic status was classified using the B. G. Prasad Scale (2023) [20]. Adequate birth spacing since the last pregnancy was defined as an inter-pregnancy interval of at least three years [15]. HRP was defined based on PMSMA criteria [2], including severe anaemia (haemoglobin < 7 g/dL), hypertensive disorders, gestational diabetes mellitus, hypothyroidism, seropositivity for HIV/syphilis, extremes of maternal age (<20 or >35 years), multiple pregnancy, malpresentation, previous CS, placental disorders, previous adverse pregnancy outcomes, Rh incompatibility, and other systemic illnesses. Regarding pregnancy registration and delivery, public facilities were defined as those owned and operated by the Government or Municipal Corporation, while private facilities were those owned and operated by private entities such as individuals, companies, or non-profit organisations [21]. Preterm delivery was defined as delivery before 37 completed weeks [15]. Early initiation of breastfeeding was defined as initiation within one hour of birth [15]. Maternal morbidity was considered if the woman experienced at least one complication such as postpartum haemorrhage, fever, urinary complaints, respiratory distress, seizures, or Intensive Care Unit (ICU) admission [5]. Neonatal morbidity included conditions such as birth asphyxia, respiratory distress, Neonatal Intensive Care Unit (NICU) admission, jaundice requiring phototherapy, infections, or congenital anomalies [10].

Data collection instruments and techniques

A Case Record Form was developed as per the study objectives and subsequently subjected to validation by experts (see Appendix 1). It consisted of four key sections: sociodemographic details, events of pregnancy, assessment for HRP, and details of delivery. The eligible women were visited at their homes, where the purpose of the study was clearly explained to them. Written informed consent was obtained from all participants. The process of administering informed consent, conducting the interview, and checking the medical records would take approximately 20-30 minutes. To ensure data integrity, the completeness of the information gathered was verified before leaving the data collection site.

Data management and statistical analysis

The anonymised dataset was entered and analysed using R version 4.5.2 (R Foundation for Statistical Computing, Vienna, Austria). Associations between categorical variables were assessed using the Chi-square or Fisher’s test, as applicable, and a p-value of less than 0.05 was considered statistically significant. The Wilcoxon signed-rank test was used to compare the median OOPE and length of stay across risk categories. Bivariate analysis was conducted to identify sociodemographic and obstetric factors associated with HRP, and variables with a p-value <0.05 were subsequently included in the multivariable logistic regression model.

Ethical considerations

Ethical approval for the study was obtained from the Institutional Ethics Committee-II, Relating to Biomedical and Health Research (BHR), Seth GS Medical College and KEM Hospital, Mumbai; Ref. No. IEC(II)/OUT/66/2023, dated February 1, 2023. All the study participants gave informed consent, and privacy and confidentiality were maintained at all stages of the study. All personal identifiers were removed before analysis to ensure data anonymity.

## Results

A total of 292 recently delivered women were included in the study. Of these, 56.5% (n = 165) of women had an HRP, with 39.5% (n = 115) having a single high-risk factor, while 17.1% (n = 50) had multiple high-risk factors (Table 1).

**Table 1:** Prevalence, stratification, and distribution of high-risk pregnancies (n = 292) *A total cannot be calculated because 50 participants had more than one high-risk pregnancy condition. The percentage of each condition is expressed as its proportion out of 292; **Other medical disorders include 2 women with seizure disorders and 1 woman with heart disease. CS: Caesarean Delivery

Variable	Frequency (N)	Percentage (%)
High-risk pregnancy		
Yes	165	56.6%
No	127	43.4%
Total	292	100%
Stratification of high-risk pregnancies		
Single high-risk	115	39.5%
Multiple high-risk	50	17.1%
Total	165	56.6%
Distribution of risk factors among high-risk pregnancies*		
Severe anaemia	40	24.2%
Previous CS delivery	40	24.2%
Previous abortion	35	21.2%
Hypertensive disorder	28	17.0%
Age >35 years	21	12.7%
Thyroid disorder	20	12.1%
Malpresentation	15	9.1%
Diabetes mellitus	9	5.5%
Rh incompatibility	7	4.2%
Antepartum haemorrhage	7	4.2%
Age <20 years	6	3.6%
History of stillbirth	6	3.6%
History of preterm birth	5	3.0%
Other medical disorders**	3	1.8%

The distribution of risk factors among women with HRPs showed that severe anaemia and previous caesarean delivery were the most frequent risk factors, each accounting for 24.2% (n = 40) of the total sample, followed by previous abortion at 21.2% (n = 35) and hypertensive disorders at 17.0% (n = 28). Advanced maternal age (>35 years) and thyroid disorders were observed in 12.7% (n = 21) and 12.1% (n = 20) of cases, respectively. Malpresentation was present in 9.1% (n = 15), while diabetes mellitus, Rh incompatibility, and antepartum haemorrhage were noted in 5.5% (n = 9), 4.2% (n = 7), and 4.2% (n = 7), respectively. Age <20 years and history of stillbirth each contributed 3.6% (n = 6), whereas history of preterm birth accounted for 3.0% (n = 5).

Table 2 presents the distribution of HRPs as per the sociodemographic characteristics of participants. The majority of participants were aged 20-24 years (n = 111, 38.0%) and 25-29 years (n = 96, 32.9%), with 6 (2.1%) adolescent pregnancies (<20 years) and 21 pregnancies (7.2%) with advanced maternal age (≥35 years). Age was significantly associated with HRP (p < 0.001), with high-risk women more evenly distributed across 20-24 years (n = 47, 28.5%), 25-29 years (n = 49, 29.7%), and 30-34 years (n = 42, 25.5%). In contrast, among non-HRPs, half of the women belonged to the 20-24 years group (n = 64, 50.4%), while 37.0% (n = 47) were aged 25-29 years, and only 12.6% (n = 16) were between 30 and 34 years of age. Regarding religion, Muslims constituted the majority of the study population (n = 234, 80.1%), followed by Hindus (n = 58, 19.9%) and Christians (n = 3, 1.0%). Most women had completed five to nine years of schooling (n = 115, 39.4%), followed by 70 (24.0%) with more than 12 years of schooling. As per the B.G. Prasad Scale (2023), 101 (34.6%) women belonged to the middle class (III), followed by 135 (46.2%) women in the lower middle (IV) and 56 (19.2%) women in the lower (V) classes. Religion, education, and socioeconomic status were not significantly associated with HRP (p = 0.09, p = 0.07, and p = 0.68, respectively), showing similar distributions across both groups. In contrast, type of family was significantly associated with HRP status (p = 0.02), with a higher proportion of HRPs among women from nuclear families (n = 88, 53.3% vs. n = 49, 38.6%), while non-HRPs were more common in joint families (n = 78, 61.4%).

**Table 2:** Distribution of high-risk pregnancies as per the sociodemographic characteristics (N = 292) *Chi-square Test; **The categories “< 20 years” and “≥ 35 years” were excluded while applying the test of significance, as extremes of maternal age were part of the criteria used to classify high-risk pregnancy.

Variable	High-risk pregnancy (N = 165); n (%)	Non-high risk pregnancy (N = 127); n (%)	Total (N = 292); n (%)	Test	p-value
Age group **					
< 20 years	6 (3.6%)	0	6 (2.1%)	^𝛘^^2^ = 13.87, df = 2	<0.001*
20-24 years	47 (28.5%)	64 (50.4%)	111 (38.0%)		
25-29 years	49 (29.7%)	47 (37.0%)	96 (32.9%)		
30-34 years	42 (25.5%)	16 (12.6%)	58 (19.9%)		
≥ 35 years	21 (12.7%)	0	21 (7.2%)		
Total	165 (56.5%)	127 (43.5%)	292 (100%)		
Religion					
Muslim	126 (76.4%)	108 (85%)	234 (80.1%)	^𝛘^^2^ = 4.77, df = 2	0.09*
Hindu	36 (21.8%)	19 (15%)	58 (19.9%)		
Christian	3 (1.8%)	0	3 (1.0%)		
Total	165 (56.5%)	127 (43.5%)	292 (100%)		
Years of schooling					
No schooling	20 (12.1%)	5 (3.9%)	25 (8.6%)	^𝛘^^2^ = 8.50, df = 4	0.07*
< 5 years	13 (7.9%)	13 (10.2%)	26 (8.9%)		
5 to 9 years	65 (39.4%)	50 (39.4%)	115 (39.4%)		
10 to 11 years	26 (15.8%)	30 (23.6%)	56 (19.2%)		
≥ 12 years	41 (24.8%)	29 (22.8%)	70 (24.0%)		
Total	165 (56.5%)	127 (43.5%)	292 (100%)		
Socioeconomic status (as per B.G. Prasad classification 2023)					
Middle (III)	55 (33.3%)	46 (36.2%)	101 (34.6%)	^𝛘^^2^ = 0.79, df = 2	0.68*
Lower Middle (IV)	80 (48.5%)	55 (43.3%)	135 (46.2%)		
Lower (V)	30 (18.2%)	26 (20.5%)	56 (19.2%)		
Total	165 (56.5%)	127 (43.5%)	292 (100%)		
Type of family					
Nuclear	88 (53.3%)	49 (38.6%)	137 (46.9%)	^𝛘^^2^ = 5.69, df = 1	0.02*
Joint	77 (46.7%)	78 (61.4%)	155 (53.1%)		
Total	165 (56.5%)	127 (43.5%)	292 (100%)		

Table 3 presents the distribution of HRPs by obstetric characteristics. Parity showed a significant association with HRP (p = 0.01), with a higher proportion of HRP among multiparous women than among primiparous women (n = 98, 59.4% vs. n = 67, 40.6%). A significant association was also observed with the number of living children (p = 0.01), with a greater proportion of women having two or more children in the high-risk group compared to the non-high-risk group. Among multiparous women, adequate spacing was reported more commonly (p = 0.01) in HRPs (n = 70, 71.4%) compared to non-HRPs (n = 32, 58.2%). The place of pregnancy registration was also significantly associated with HRP (p = 0.02). Overall, public sector registration was more common (n = 170, 58.2%), but a higher proportion of HRPs were registered in private facilities (n = 75, 45.5%) compared to non-HRPs (n = 38, 29.9%). Nine pregnancies (3.1%) were unregistered. Regarding the trimester of registration, 71.7% of women (n = 203) had registered in the first trimester of pregnancy, with no significant difference across both groups (p = 0.81).

**Table 3:** Distribution of high-risk pregnancies as per the obstetric characteristics (N = 292) † N = 153 women because the inter-pregnancy interval can be calculated only in multiparous women; ‡ N = 283 women because there were 283 registered pregnancies; *Chi-square Test; **Fisher’s Exact Test.

Variable	High-risk pregnancy (N = 165); n (%)	Non-high risk pregnancy (N = 127); n (%)	Total (N = 292); n (%)	Test	p-value
Parity					
Primipara	67 (40.6%)	72 (56.7%)	139 (47.6%)	^𝛘^^2^ = 6.81, df = 1	0.01*
Multipara	98 (59.4%)	55 (43.3%)	153 (52.4%)		
Total	165 (56.5%)	127 (43.5%)	292 (100%)		
Number of living children					
1	67 (40.6%)	72 (56.7%)	139 (47.6%)	Fisher’s Exact Test	0.01**
2	58 (35.2%)	33 (26.0%)	91 (31.2%)		
3	29 (17.6%)	19 (15.0%)	48 (16.4%)		
≥ 4	11 (6.7%)	3 (2.4%)	14 (4.8%)		
Total	165 (56.5%)	127 (43.5%)	292 (100%)		
Inter-pregnancy interval or spacing †					
Adequate	70 (71.4%)	32 (58.2%)	102 (66.7%)	^𝛘^^2^ = 10.05, df = 2	0.01*
Inadequate	28 (28.6%)	23 (41.8%)	51 (33.3%)		
Total	98 (59.4%)	55 (43.3%)	153 (100%)		
Place of pregnancy registration					
Public	86 (52.1%)	84 (66.1%)	170 (58.2%)	Fisher’s Exact Test	0.02**
Private	75 (45.5%)	38 (29.9%)	113 (38.7%)		
Not registered	4 (2.4%)	5 (3.9%)	9 (3.1%)		
Total	165 (56.5%)	127 (43.5%)	292 (100%)		
Trimester of registration ^‡^					
First	113 (70.2%)	90 (73.8%)	203 (71.7%)	Fisher’s Exact Test	0.81**
Second	42 (26.1%)	28 (23.0%)	70 (24.7%)		
Third	6 (3.7%)	4 (3.3%)	10 (3.5%)		
Total	161 (56.9%)	122 (43.1%)	283 (100%)		

Table 4 presents the bivariate and multivariate analyses of sociodemographic and obstetric factors associated with HRP. In the bivariate analysis, women aged 30-34 years had significantly higher odds of HRP (crude OR: 3.57, 95% CI: 1.82-7.27; p < 0.001) as compared to those aged 20-24 years. Women with no schooling had four times higher odds of HRP than those with <5 years of schooling (crude OR: 4.00, 95% CI: 1.20-15.03; p = 0.03). Nuclear family type was associated with higher odds of HRP compared with joint family type (crude OR: 1.82, 95% CI: 1.14-2.92; p = 0.01). Multiparous women had significantly higher odds of HRP than primiparous women (crude OR: 1.78, 95% CI: 1.29-2.49; p < 0.001). Registration at a private facility was also significantly associated with HRP (crude OR: 1.97, 95% CI: 1.35-2.94; p < 0.001), while religion, inter-pregnancy interval, and socioeconomic status did not show an independent association in the bivariate analysis. On multivariable analysis, only multiparity (adjusted OR: 1.85, 95% CI: 1.07-3.20; p = 0.03) remained an independent predictor of HRP after adjustment, while women aged 25-29 years demonstrated significantly lower adjusted odds of HRP (adjusted OR: 0.54, 95% CI: 0.30-0.96; p = 0.04).

**Table 4:** Bivariate and multivariate analyses of sociodemographic and obstetric factors associated with high-risk pregnancy

Variable	Category	Crude OR (95% CI)	p-value	Adjusted OR (95% CI)	p-value
Age group	20-24 years	1.0 (Ref)	-	1.0 (Ref)	-
	25-29 years	1.42 (0.82-2.47)	0.21	0.54 (0.30-0.96)	0.04
	30-34 years	3.57 (1.82-7.27)	<0.001	1.44 (0.71-2.97)	0.30
Religion	Muslim	1.0 (Ref)	-	-	-
	Non-Muslim	0.99 (0.55-1.82)	0.98	-	-
Years of schooling	No schooling	4.00 (1.20-15.03)	0.03	3.35 (0.85-14.37)	0.09
	<5 years	1.0 (Ref)	-	1.0 (Ref)	-
	5-9 years	1.30 (0.55-3.07)	0.55	1.56 (0.53-4.54)	0.41
	10-11 years	0.87 (0.34-2.21)	0.76	0.85 (0.27-2.60)	0.78
	>12 years	1.41 (0.57-3.52)	0.45	1.88 (0.60-5.82)	0.27
Type of family	Joint	1.0 (Ref)	-	1.0 (Ref)	-
	Nuclear	1.82 (1.14-2.92)	0.01	1.72 (0.99-3.00)	0.05
Socioeconomic status	Middle (III)	1.0 (Ref)	-	-	-
	Lower middle (IV)	1.21 (0.72-2.05)	0.46	-	-
	Lower (V)	0.96 (0.50-1.86)	0.92	-	-
Parity	Primipara	1.0 (Ref)	-	1.0 (Ref)	-
	Multipara	1.78 (1.29-2.49)	<0.001	1.85 (1.07-3.20)	0.03
Inter-pregnancy interval	Inadequate	1.0 (Ref)	-	-	-
	Adequate	1.80 (0.90-3.60)	0.10	-	-
Place of registration	Public facility	1.0 (Ref)	-	1.0 (Ref)	-
	Private facility	1.97 (1.35-2.94)	<0.001	1.46 (0.86-2.49)	0.16

Table 5 shows delivery outcomes stratified by the type of pregnancy. HRPs had a significantly greater proportion (p = 0.04) of preterm deliveries than non-HRPs (n = 20, 12.1% vs. n = 6, 4.7%). Regarding the mode of delivery, CS was considerably more common (p < 0.001) in the high-risk group than in the non-high-risk group (n = 92, 55.8% vs. n = 30, 23.6%). Place of delivery did not show a significant association (p = 0.15), with most deliveries in both groups occurring at public facilities (n = 176, 60.3%). Overall, 32.9% (n = 96) of babies were low birth weight, with no significant difference between the groups (p = 0.43). Early initiation of breastfeeding was significantly lower among HRPs, being reported in 49.1% (n = 81) compared with 80.3% (n = 102) among non-HRPs (p < 0.001). Maternal morbidity was higher in the high-risk group (n = 32, 19.4% vs. n = 14, 11.0%), although this difference was not statistically significant (p = 0.07). Neonatal morbidity was significantly more common among HRPs than non-HRPs (n = 64, 38.8% vs. n = 27, 21.3%) (p = 0.002). Additionally, the median OOPE during delivery was significantly higher among HRPs than non-HRPs (INR 6,000 vs. INR 4,000, p = 0.009), and the median length of hospital stay was also longer in the high-risk group (4 days vs. 3 days, p < 0.001).

**Table 5:** Delivery characteristics stratified by the type of pregnancy (N = 292) † For analysis, deliveries occurring at home and in transit were excluded, as the comparison was restricted to institutional deliveries to assess the association between the sector of delivery (public vs. private) and high-risk pregnancy status; *Chi-square Test, **Fisher’s Exact Test, ***Wilcoxon signed rank test. OOPE: Out-of-Pocket Expenditure

Variable	High-risk pregnancy (N = 165); n (%)	Non-high risk pregnancy (N = 127); n (%)	Total (N = 292); n (%)	Test	p-value
Time of delivery					
Preterm	20 (12.1%)	6 (4.7%)	26 (8.9%)	^𝛘^^2^ = 6.45, df = 2	0.04*
Term	132 (80.0%)	115 (90.6%)	247 (84.6%)		
Post-term	13 (7.9%)	6 (4.7%)	19 (6.5%)		
Total	165 (56.5%)	127 (43.5%)	292 (100%)		
Mode of delivery					
Normal vaginal	73 (44.2%)	97 (76.4%)	170 (58.2%)	^𝛘^^2^ = 29.16, df = 1	<0.001*
Caesarean section	92 (55.8%)	30 (23.6%)	122 (41.8%)		
Total	165 (56.5%)	127 (43.5%)	292 (100%)		
Place of delivery †					
Public facility	94 (57.0%)	82 (64.6%)	176 (60.3%)	^𝛘^^2^ = 2.09, df = 1	0.15*
Private facility	70 (42.4%)	41 (32.3%)	111 (38.0%)		
Home	1 (0.6%)	3 (2.4%)	4 (1.4%)		
In transit	0	1 (0.8%)	1 (0.3%)		
Total	165 (56.5%)	127 (43.5%)	292 (100%)		
Birth weight of the baby					
Low	54 (32.7%)	42 (33.1%)	96 (32.9%)	Fisher’s Exact Test	0.43**
Normal	108 (65.5%)	85 (66.9%)	193 (66.1%)		
Overweight	3 (1.8%)	0	3 (1.0%)		
Total	165 (56.5%)	127 (43.5%)	292 (100%)		
Early initiation of breastfeeding					
Yes	81 (49.1%)	102 (80.3%)	183 (62.7%)	^𝛘^^2^ = 28.59, df = 1	<0.001*
No	84 (50.9%)	25 (19.7%)	109 (37.3%)		
Total	165 (56.5%)	127 (43.5%)	292 (100%)		
Maternal morbidity					
Yes	32 (19.4%)	14 (11%)	46 (15.8%)	^𝛘^^2^ = 3.18, df = 1	0.07*
No	133 (80.6%)	113 (89%)	246 (84.2%)		
Total	165 (56.5%)	127 (43.5%)	292 (100%)		
Neonatal morbidity					
Yes	64 (38.8%)	27 (21.3%)	91 (31.2%)	^𝛘^^2^ = 9.48, df = 1	0.002*
No	101 (61.2%)	98 (78.7%)	199 (68.8%)		
Total	165 (56.5%)	127 (43.5%)	292 (100%)		
OOPE during delivery, median (IQR)	6000 (34,250)	4000 (18,000)	5000 (26,500)	V = 11972	0.009***
Length of stay, median (IQR)	4 (2)r	3 (2)	3 (3)	V = 13544	<0.001***

## Discussion

The present study assessed the prevalence of HRPs among 292 recently delivered women in an urban slum community and the sociodemographic factors, obstetric factors, and delivery characteristics associated with them.

The prevalence of HRP in this study was 56.5% (n = 165), with 39.5% (n = 115) having a single risk factor and 17.1% (n = 50) having multiple risk factors. This is comparable to NFHS-5 estimates using PMSMA criteria, which reported a national prevalence of 49.5% and 47.7% in Maharashtra, with a similar distribution of single (n = 115, 33%) and multiple (n = 50, 16.4%) risk factors [11]. However, the prevalence observed in this study is higher than that reported in several facility-based studies in India, such as 12.7% by Dahiya et al. [22] in Haryana, 28.6% by Dwivedi et al. [23] in Jabalpur, 33.6% by Jadhao et al. [24] in Nagpur, 34.3% by Mogan et al. [25] in Delhi, and 40.5% by Prajapati et al. [26] in Uttar Pradesh, many of which were conducted in rural settings. The prevalence also exceeds estimates from other countries such as Nepal (ranging from 14% to 33%) [5,9,10], 19.1% by Powell et al. [8] in Mexico, 26.4% by Jemila et al. [7] in Ethiopia, and 33.1% by Sylvie et al. [4] in the Democratic Republic of Congo. Nevertheless, higher prevalence has been reported in certain settings such as 75% by Farajnezhad et al. [27] in a peri-urban area of Iran and 63.3% by Kamal Hafez et al. [28] in Saudi Arabia, owing to epidemiological transition and the rising burden of NCDs. The high prevalence in the present study may be attributed to the urban slum setting and the inclusion of a wide range of obstetric and medical risk factors as per the PMSMA criteria.

In the present study, severe anaemia and previous CS were the most common risk factors (n = 40, 24.2% each), followed by previous abortion (n = 35, 21.2%) and hypertensive disorders (n = 28, 17.0%), consistent with existing literature [4,6,7,9,10,22,26,28]. However, the prevalence of severe anaemia was much higher than NFHS-5 estimates (1.4% and 2.4% in pregnant and lactating women, respectively) [15] and other Indian studies, such as 1.4% each by Mogan et al. [25] and Kuppusamy et al. [11], 1.5% by Dwivedi et al. [23], and 6.3% by Ashrita and Sarvagnya [29], highlighting persistent micronutrient deficiencies among women in urban slums. Previous CS rates were comparable to or higher than those in other Indian studies: 3% by Prajapati et al. [26], 14.5% by Jadhao et al. [24], 16.4% by Kuppusamy et al. [11], 19.1% by Mogan et al. [25], 22% by Dwivedi et al. [23], and 31% by Dahiya et al. [22]. This reflects the ongoing rise in CS rates in India, creating a cycle of repeat HRPs [14,17]. Previous abortion and hypertensive disorders were also common, aligning with both national and global evidence highlighting their significant contribution to HRPs (ranging from 12.4% to 35% for previous abortion and 0.3% to 36% for hypertensive disorders) [6-8,10,11,23-25,29].

Advanced maternal age (≥35 years) was observed in 12.7% of cases (n = 21), higher than that reported in Indian studies such as 2.2% by Kuppusamy et al. [11] and 2.6% by Dwivedi et al. [23]. However, other urban settings have reported higher proportions, such as 19.8% among women in the DRC by Sylvie et al. [4] and 21% in urban Brazil by Sampaio et al. [6], likely reflecting higher maternal age in more urbanised and developed settings. Hypothyroidism was a risk factor in 12.1% of cases (n = 20), which is higher than that reported in other Indian studies such as Shrestha et al. (5.3%), Kuppusamy et al. (2.3%), and Dwivedi et al. (9%) [10,11,23]. In contrast, hypothyroidism has also emerged as a leading risk factor in some settings, such as Dahiya et al. (13.1%) and Mogan et al. (43.7%), indicating variability across populations and diagnostic practices [22,25]. Other risk factors included malpresentation (n = 15, 9.1%), diabetes mellitus (n = 9, 5.5%), Rh incompatibility (n = 7, 4.2%), antepartum haemorrhage (n = 7, 4.2%), and adolescent pregnancy (<20 years) (n = 6, 3.6%), with relatively lower prevalence, consistent with previous studies where these conditions were reported as less frequent contributors to HRPs but remain clinically significant due to their potential impact on maternal and neonatal health [9,10,23,25,29].

Regarding sociodemographic characteristics, age showed a significant association with HRP on bivariate analysis, with 3.57 times higher odds of HRP in women aged 30-34 years. However, in adjusted analysis, the association was no longer significant (p = 0.30), and women aged 25-29 years had significantly lower odds of HRP (p = 0.04), suggesting that the crude association may be confounded by factors such as parity. Also, extreme ages were excluded from statistical testing because they were part of the HRP definition, which may have influenced the strength of the observed association. Several studies have demonstrated increasing maternal age as an important risk factor due to its association with chronic conditions such as hypertension, diabetes, chromosomal abnormalities, preterm births, and CS deliveries [12]. The mean age in our study was 26.7 (±4.65) years, which is slightly higher than in other Indian studies such as Jadhao et al. (24.4 years), Dwivedi et al. (24.5 years), Mogan et al. (24.0 years), and Prajapati et al. (25 years), but slightly lower than in other global urban settings such as Sylvie et al. (28.8 years) and Sampaio et al. (28.4 years), possibly reflecting a transitional pattern of increasing age of childbearing due to the influence of urbanisation [4,6,23-26]. Although nuclear family type was associated with 1.82 times higher odds of HRP in the unadjusted analysis, this association lost significance after adjustment. While several studies report strong associations with education, socioeconomic status, and religion [7,11,23,26], these factors were not significantly associated with HRP in our study, possibly due to the relative homogeneity of the study population.

Regarding obstetric characteristics, multiparity was significantly associated with HRP and remained significant even on multivariate analysis. This finding is consistent with several Indian studies, which suggest the possible cumulative effect of previous pregnancies, including prior CS, abortions, and other complications in multiparous women [29]. Similarly, a higher number of living children was associated with HRP, further supporting the association of increasing parity with pregnancy risk. However, adequate inter-pregnancy spacing was more common among HRPs, which may be due to women with prior adverse obstetric outcomes consciously delaying subsequent pregnancies but continuing to be classified as high-risk due to their adverse obstetric history [10,11,23]. Overall, 71.7% of women (n = 203) registered in the first trimester of pregnancy, with no significant differences across risk categories. Though encouraging, this is lower than NFHS-5 estimates (85.2% and 89.5% for India and Maharashtra, respectively) [15], possibly due to the urban slum setting of the study. The place of pregnancy registration was significantly associated with HRP on bivariate analysis, with women registered in private facilities having nearly twice the odds of being high-risk compared to those in public facilities. However, this association was not retained on multivariable analysis, suggesting that it may be explained by underlying factors such as maternal age, parity, and prior obstetric history rather than an independent effect of the place of registration.

Regarding delivery characteristics, HRPs were associated with a significantly higher proportion of preterm deliveries (p = 0.04), consistent with the established link between obstetric complications and early delivery [5,12]. CS rates were also significantly higher (p < 0.001), reflecting both clinical indications and precautionary practices reported in previous studies [10,24]. Although earlier studies have shown an association between HRP and low birth weight [10,24], this was not observed in the present study (p = 0.43), with a similar distribution of low birth weight babies across both groups (n = 54, 32.7% in the HRP group vs. n = 42, 33.1% in the non-HRP group). Neonatal morbidity was significantly higher among HRPs (p = 0.002), aligning with findings from Shrestha et al. [10]. Maternal morbidity was also higher in the high-risk group, although not statistically significant (p = 0.07), in contrast to studies that have demonstrated a stronger association [5,10]. Early initiation of breastfeeding was significantly lower among HRPs (p < 0.001), possibly due to higher CS deliveries and neonatal complications, consistent with previous literature such as Salama et al. [30], which reports higher rates of breastfeeding failure among HRPs. Furthermore, HRPs were associated with significantly higher OOPE (p = 0.009) and longer hospital stay (p < 0.001), indicating an increased healthcare and financial burden. These findings highlight the importance of early identification and effective management of HRPs to reduce maternal and neonatal complications and associated costs.

The present study has certain limitations. Being a cross-sectional design, causal relationships between risk factors and HRP cannot be established. Information on some variables was self-reported or based on available records, which may be subject to recall bias and incomplete documentation. Potential selection bias is also acknowledged, arising from register-based sampling. The study is limited by the exclusion of stillbirth outcomes, potentially underrepresenting the burden of adverse pregnancy outcomes. The use of PMSMA criteria for HRP classification is programmatically relevant in India but not internationally standardised, potentially limiting comparability with studies using other risk classification frameworks. The classification of HRP depended on documented conditions, and underdiagnosis of conditions such as hypothyroidism or anaemia may have led to misclassification. Some variables, such as birth weight and complications, may also have been influenced by the quality of care received, which was not independently assessed.

Our findings highlight the need to strengthen early identification and management of HRPs at the community level in our study area, with special focus on multiparous women and those of increasing maternal age. Targeted interventions to address modifiable risk factors, particularly anaemia, should be prioritised through assessment of the implementation of existing programmes such as Anaemia Mukt Bharat, including assessment of the availability of and adherence to iron and folic acid supplementation. Preventive strategies should also begin earlier in the life course, with the strengthening of nutritional interventions during childhood and adolescence to reduce the burden of anaemia before pregnancy. The high proportion of CS warrants further evaluation to assess the appropriateness of indications and identify potentially avoidable procedures. Efforts should also be made to promote early initiation of breastfeeding, especially among high-risk mothers, through enhanced counselling and lactational support.

## Conclusions

HRPs were highly prevalent in the study population, affecting more than half of the women, with severe anaemia and previous CS emerging as the most common risk factors. HRP was significantly associated with increasing maternal age and multiparity and was linked to a higher rate of CS, poorer maternal and neonatal indicators, and increased healthcare costs. These findings highlight the need for strengthening early identification through routine screening, addressing modifiable risk factors such as anaemia across the life course, evaluating the appropriateness of rising CS rates, and improving access to affordable, quality maternal healthcare to reduce the burden of HRPs in the study area.

## Appendices

Appendix 1: Case record form

Date:

Participant ID:

A. Sociodemographic details

1) Age (in years)

2) Religion: ☐ Hindu ☐ Muslim ☐ Christianity ☐ Any other

3) Number of years of schooling:

4) Employment: ☐ Employed ☐ Unemployed

5) If employed, Occupation: ____________ Monthly income: ____________

6) Type of family: ☐ Nuclear ☐ Joint ☐ Other

7) Number of family members:

8) Highest education of Head of family: ☐ Professional ☐ Graduate ☐ Intermediate or Diploma ☐ HSC ☐ Middle School ☐ Primary School ☐ Illiterate

9) Occupation of Head of family: ☐ Professional ☐ Semi-professional ☐ Clerical or shop owner ☐ Skilled ☐ Semi-skilled ☐ Semi-skilled ☐ Unemployed

10) Marital status - ☐ Married ☐ Divorced ☐ Widowed

11) Married since: _____ (years)

12) Number of children:

13) Interval since last pregnancy (in months):

B. Events of pregnancy

1) Was your pregnancy registered? ☐ Yes ☐ No

2) If yes, where was the pregnancy registered? ☐ Public facility ☐ Private facility And when: ☐ First trimester ☐ Second trimester ☐ Third trimester

3) Was a first-trimester USG performed? ☐ Yes ☐ No

4) Total number of antenatal visits -

5) Number of doses of Td: ☐ 1 ☐ 2 ☐ Booster

6) Were you provided IFA tablets for 180 days? ☐ Yes ☐ No If yes, how long did you consume them for?___________ (months)

If consumed for less than 6 months, provide reasons:

7) Were the following things done?

a) Date of LMP was recorded: ☐ Yes ☐ No

b) Hb checked and recorded: ☐ Yes ☐ No

c) Weight recorded: ☐ Yes ☐ No

d) BP checked and recorded: ☐ Yes ☐ No

e) Abdominal exam done: ☐ Yes ☐ No

f) HIV test done and reported: ☐ Yes ☐ No

g) Urine exam reported: ☐ Yes ☐ No

C. Assessment of high-risk pregnancy

1) Hb <7: ☐ Yes ☐ No

2) Blood transfusion received during pregnancy ☐ Yes ☐ No

3) Taking treatment for hypertension ☐ Yes ☐ No

4) History of seizures ☐ Yes ☐ No

5) Admission due to raised BP during pregnancy ☐ Yes ☐ No

6) BP > 140/90 mm Hg documented in medical records? ☐ Yes ☐ No

7) Taking treatment for diabetes ☐ Yes ☐ No

8) If yes, taking insulin ☐ Yes ☐ No

9) Thyroid disorder: ☐ Yes ☐ No

10) Heart disease: ☐ Yes ☐ No

11) HIV status: ☐ Yes ☐ No ☐ Not known

12) Presentation of foetus: ☐ Vertex ☐ Face/Brow ☐ Shoulder ☐ Breech ☐ Other

13) Any bleeding in the third trimester: ☐ Yes ☐ No

14) Placenta previa or abruption documented in records ☐ Yes ☐ No

15) Rh incompatibility ☐ Yes ☐ No

16) Multiple pregnancy ☐ Yes ☐ No

17) Any of the following during pregnancy? ☐ Trauma ☐ Jaundice ☐ TB ☐ Malaria ☐ No

18) If multipara - were any of the following conditions present during previous childbirth: ☐ Caesarean section ☐ Stillbirth ☐ Abortion ☐ Preterm birth ☐ Ectopic pregnancy

D. Maternal and neonatal outcomes

1) Time of delivery: ☐ Preterm ☐ Term ☐ Post-term

2) Place of delivery: ☐ Home ☐ Health facility ☐ In transit

3) Mode of delivery: ☐ Normal vaginal ☐ Emergency CS ☐ Elective CS

4) If LSCS, what was the indication(s) documented?

5) Sex of the baby: ☐ Male ☐ Female

6) Birth weight of baby: ______ kg

7) Any maternal morbidity:

8) Any neonatal morbidity:

9) NICU admission required? ☐ Yes ☐ No

10) Early initiation of breastfeeding: ☐ Yes ☐ No

11) Birth immunisation: ☐ BCG ☐ OPV ☐ Hepatitis B

12) Length of hospital stay: _____ days
